# Genomic Analysis of Sindbis Virus Reveals Uncharacterized Diversity within the Australasian Region, and Support for Revised SINV Taxonomy

**DOI:** 10.3390/v16010007

**Published:** 2023-12-20

**Authors:** Alice Michie, Timo Ernst, Alyssa T. Pyke, Jay Nicholson, John S. Mackenzie, David W. Smith, Allison Imrie

**Affiliations:** 1 School of Biomedical Sciences, University of Western Australia, Nedlands, WA 6009, Australia; alice.michie@uwa.edu.au (A.M.); timo.ernst@uwa.edu.au (T.E.); 2 Department of Health, Public Health Virology Laboratory, Forensic and Scientific Services, Queensland Government, Coopers Plains, QLD 4108, Australia; alyssa.pyke@health.qld.gov.au; 3 Environmental Health Directorate, Department of Health, Perth, WA 6000, Australia; jay.nicholson@health.wa.gov.au; 4 PathWest Laboratory Medicine Western Australia, Nedlands, WA 6009, Australia; j.mackenzie@curtin.edu.au (J.S.M.); david.smith@health.wa.gov.au (D.W.S.); 5 School of Chemistry and Molecular Biosciences, University of Queensland, St. Lucia, QLD 4072, Australia; 6 Faculty of Health Sciences, Curtin University, Bentley, WA 6102, Australia

**Keywords:** Sindbis virus, Argyle virus, Thomson’s Lake virus, alphavirus, *Togaviridae*, arbovirus, Australia, species demarcation, phylogeny, Sindbis virus genotypes

## Abstract

Sindbis virus (SINV) is a widely dispersed mosquito-borne alphavirus. Reports of Sindbis disease are largely restricted to northern Europe and South Africa. SINV is frequently sampled in Australian mosquito-based arbovirus surveillance programs, but human disease has rarely been reported. Molecular epidemiological studies have characterized six SINV genotypes (G1–G6) based on E2 gene phylogenies, mostly comprising viruses derived from the African–European zoogeographical region and with limited representation of Australasian SINV. In this study, we conducted whole genome sequencing of 66 SINV isolates sampled between 1960 and 2014 from countries of the Australasian region: Australia, Malaysia, and Papua New Guinea. G2 viruses were the most frequently and widely sampled, with three distinct sub-lineages defined. No new G6 SINV were identified, confirming geographic restriction of these viruses to south-western Australia. Comparison with global SINV characterized large-scale nucleotide and amino acid sequence divergence between African–European G1 viruses and viruses that circulate in Australasia (G2 and G3) of up to 26.83% and 14.55%, respectively, divergence that is sufficient for G2/G3 species demarcation. We propose G2 and G3 are collectively a single distinct alphavirus species that we name Argyle virus, supported by the inapparent or mild disease phenotype and the higher evolutionary rate compared with G1. Similarly, we propose G6, with 24.7% and 12.61% nucleotide and amino acid sequence divergence, is a distinct alphavirus species that we name Thomson’s Lake virus.

## 1. Introduction

SINV is the most widely distributed of the alphaviruses with virus isolations reported from multiple countries across Europe, Asia, Africa, and the wider Australasian region, including Papua New Guinea (PNG), New Zealand, and Australia. The prototype SINV (strain AR339) was sampled in the village of Sindbis in Egypt in 1953 from a mixed pool of *Culex pipiens* and *Cx. univittatus* mosquitoes [1,2]. SINV was first isolated from symptomatic humans in Uganda in 1961 and was recognized as a cause of arthritic disease in South Africa in 1963 [3].

Apparent SINV infection is associated with an arthritogenic syndrome that is often accompanied by myalgia, malaise, headache, fever, and rash [4,5,6]. SINV disease primarily occurs in northern Europe with periodic disease outbreaks reported in South Africa [7,8]. In Australia, serological evidence suggests sub-clinical SINV infections have occurred in many areas across the country [9,10,11,12,13]; however, just two clinical cases have been documented to date [9,14]. Two other alphaviruses, Ross River virus (RRV) and Barmah Forest virus (BFV), circulate in Australia and are associated with classic arthritogenic disease that is similar to the SINV-associated disease reported in Europe and Africa [4,15].

Phylogenetic studies of globally sampled SINV, most based on partial gene sequence analysis [7,16,17,18,19], have primarily focused on SINV originating from Africa and Europe. Six SINV genotypes (G1–G6) have been characterized in the most recent genome-scale phylogenetic review [7]. The six genotypes demonstrate clear geographical demarcation with G1 sampling exclusive to Europe and Africa; G2 and G3 to southern and northern areas of the Australasian region, respectively; and G4 to central Asia. G5 is listed as a unique species, Whataroa virus (WHAV), in the most recent 10th ICTV report [20]. WHAV was first sampled in New Zealand in 1962, and later in south-east Australia between 1989 and 1990 [21,22]. G6, previously referred to as the ‘South-West’ (SW) genotype, has been restricted to the south-west region of Western Australia (WA) despite ample sampling of SINV throughout Australia; [16] indeed, SINV is one of the most frequently detected viruses in Australian mosquito-based surveillance programs [23]. The historical literature has detailed three major SINV groups named for the broad zoogeographical region of virus sampling—the SW, the Paleoartic/Ethiopian (PE), and the Oriental/Australasian (OA) genotypes [16,24]. The PE nomenclature encompasses G1 and G4, while OA encompasses G2 and G3.

The movement of SINV between distal geographic regions is thought to be facilitated by migratory birds travelling along trans-continental flyway routes [18,25,26]. This is based on the detection of anti-SINV antibodies in various bird species, and the genetic relatedness of virus variants sampled in distal locations, along these flyways [18,25,27,28,29].

Outbreaks of human disease have been recorded only where G1 circulates: South Africa and northern European countries, namely Sweden, Finland, and the Karelian region of north-western Russia. Disease syndromes known as Ockelbo disease, Pogosta disease, and Karelian fever, respectively [5,30,31,32], were previously regarded as being caused by unique SIN-like viruses named for the diseases they were associated with. A partial E2 phylogenetic analysis by Lundstrom et.al. (2010), however, reported there was no genetic basis for the demarcation of these viruses given their near-identical nucleotide sequence similarity [18]. SINV movement between northern Europe and South Africa was originally proposed to be facilitated by migratory birds travelling between the two distal locations and by-passing central Europe and northern Africa [25]; however, a recent genome-scale phylogenetic and evolutionary analysis of G1 indicated that SINV may have been introduced to Europe from central Africa, rather than South Africa, following a single introduction event in the 1920s [19].

Phylogenetic analysis of SINV isolates derived from Australia has been limited to a single historical partial E2 gene sequence analysis [16]. This analysis, which included reference sequences sampled from Egypt, South Africa, PNG, New Zealand, and Malaysia, defined two major phylogenetic groups that circulated in Australia—the SW and the OA, otherwise known as G6 and G2, respectively, based on the more recent nomenclature [7,16]. The SW group was sampled exclusively in the south-west region of Western Australia while the OA group was sampled Australia-wide, in PNG, and in Malaysia. Whole genome information for Australian-derived SINV is currently limited to just a single sequence for the SW prototype, SW6562, isolated from mosquitoes trapped in metropolitan Perth in 1990 [16]. The genetic diversity of SINV circulating within Australia and the wider region has not been characterized at the genome scale.

There is little whole genome information for SINV circulating beyond Africa and Europe with limited representation of the Australasian zoogeographic zone, including viruses circulating within, and proximal to, Australia. To address this, we derived complete genome sequences of Australasian-derived SINV and conducted robust phylogenetic and evolutionary analyses. These data were then used to supplement the global SINV phylogeny, to define the relatedness of Australasian SINV to SINV sampled in other zoogeographical regions, and to characterize intra-SINV divergence at the genome scale.

## 2. Materials and Methods

### 2.1. Virus Isolates

To investigate SINV diversity within the Australasian region, 66 isolates derived from PNG, Malaysia, and various locations within Australia including Western Australia (WA), Mt Isa, and the Torres Strait (TS) in Queensland (QLD) were selected for whole genome sequencing. Most isolates were from an archival arbovirus collection sampled over the course of routine mosquito-based arbovirus surveillance in WA. Viruses were isolated from 11 mosquito species, most commonly *Cx. annulirostris* (41/66 isolates). At the time of the present study, a single Australasian-derived SINV genome was publicly available: SW6562 (accession no.: AF429428), sampled in south-west WA in 1990 [33]. The final Australasian dataset was sampled over a 54-year period, 1960–2014.

For the analysis of SINV sampled globally, an additional 64 published genome sequences were accessed from the NCBI database. These viruses were sampled from 22 countries within Africa, the Middle East, northern and central Europe, and Australasia over a 63-year period (1953–2016). The genome of the 1953 Egyptian SINV prototype, AR339, was sequenced for the first time in the present study. Additional large partial genome sequences (approximately 2300 nucleotides in length) were also accessed for the analysis of previously described genotype 3 SIN viruses (NCBI accession numbers FJ744528-31).

Both datasets were subject to maximum likelihood phylogenetic reconstruction and analysis. Other sub-datasets of interest were also subject to Bayesian coalescent analyses, as described in the following sections.

### 2.2. RNA Extraction, Library Preparation, and Sequencing

Each virus selected for sequencing was amplified once on Vero cells (ATCC, CCL-81™) or C6/36 cells (ATCC, CRL-1660™) to ensure sufficient RNA for downstream sequencing. The passage histories of isolates were varied, with earlier isolates (for example, OR6) passaged at a low level in suckling mouse brain Other viruses, sampled throughout the WA surveillance program, were isolated often via low-level passage in a mosquito cell line (C6/36 cells) followed by passage in a mammalian cell line (often Vero cells or BHK-21 cells (ATCC, CCL-10™)). Sequencing was conducted as per previously described protocols [34,35]. Briefly, RNA was extracted using the Roche High Pure viral isolation kit (Roche, Basel, Switzerland) from a filtered, concentrated viral culture supernatant. The TruSeq Stranded mRNA library preparation kit (Illumina, San Diego, CA, USA) was used to generate DNA libraries as per Michie et al. 2020 [31]. The libraries were quantified and validated using the Agilent DNA 1000 kit (Integrated Sciences, Santa Clara, CA, USA) before being pooled, denatured, and sequenced on an Illumina MiSeq instrument with a MiSeq v2 (300 cycles) sequencing reagent kit.

Reads were assessed for quality using FastQC v0.11 [36] and trimmed of PhiX and adapters before being subject to *de novo* genome assembly within CLC Genomics Workbench v7.5 (QIAGEN, Hilden, Germany). Reads were mapped back to the generated contiguous sequences within Geneious v11.1 to ensure sequencing confidence [37].

For SINV isolates obtained from QLD, RNA extraction and next-generation sequencing was performed as previously described [35,38]. Briefly, RNA was extracted from 140 µL of viral culture supernatant using the QIAamp Viral RNA extraction kit (QIAGEN, Chadstone, Australia) without carrier RNA. DNase treatment of the template RNA (Heat&Run kit, ArcticZymes, Scientifix South Yarra, Victoria, Australia), first-strand (Protoscript II First Strand complementary DNA (cDNA) kit) cDNA, and second-strand cDNA synthesis was then performed using an enzyme cocktail of Escherichia coli DNA ligase, DNA polymerase I, and RNase H (first- and second-strand cDNA synthesis reagents obtained from New England Biolabs, Notting Hill, Victoria, Australia). The cDNA libraries were constructed using the Nextera XT kit and individual indices (kit A) for barcoding (Illumina, San Diego, CA, USA) according to the manufacturer’s instructions. Paired-end (2 × 150 nucleotides) massive parallel sequencing was performed using the v2 Mid-Output Kit on a NextSeq 500 machine (Illumina, San Diego, CA, USA). Raw sequence reads were processed usingGeneious R10 version 10.2.6 software [37], which included pairing of forward and reverse reads and trimming at both ends using BBduk to remove low-quality reads. Near-complete genome sequences were assembled using the rnaSPAdes de novo assembler [39] within Geneious.

### 2.3. Phylogenetic Analysis

Multiple sequence alignments of derived genome sequences were constructed for each dataset using Multiple Alignment using Fast Fourier Transform (MAFFT 7.338) within Geneious v11.1, using the default settings [40]. Prior to maximum likelihood (ML) phylogeny reconstruction, alignments were manually trimmed to remove the partial 5′ and 3′ UTRs. RAxML v8.2 was used to reconstruct the ML phylogenies with 1000 bootstrap replicates, under a GTR + Γ model [41]. The resulting ML trees were visualized and illustrated within FigTree v1.4.4 [42].

### 2.4. Coalescent Analysis

To estimate the timescale and evolutionary rates of the SINV datasets, Bayesian Markov Chain Monte Carlo (MCMC) analysis was conducted using the BEAST v1.8 package [43]. Prior to analysis, each temporally defined dataset was assessed for temporal signal using TempEst v1.5 [44].

For each dataset, a GTR + Γ + I nucleotide substitution model was assumed under an uncorrelated lognormal (UCLN) molecular clock. A minimum chain length of 1.0 × 10^8^ was used for each dataset and ran in triplicate. Independent chains were assessed for adequate convergence within Tracer v1.7 and subsequently combined using LogCombiner with a 10% burn-in [45].

Maximum clade credibility (MCC) phylogenies were reconstructed using TreeAnnotator with a 10% burn-in [43]. All MCCs were visualized and edited within FigTree v1.4. Nucleotide substitution rates as well as divergence times were estimated for each dataset, with error reported as the 95% highest probability density (95% HPD).

### 2.5. Recombination Analysis

Recombination within the dataset was queried using the RDP4 program (version 4.80). Multiple sequence alignments were analyzed using the RDP [46], Chimaera [47], Bootscan [48], 3Seq [49], GENECOV [50], SiScan [51], Phylpro [52], LARD [53], and MaxChi [54] methods under the default conditions. Justification of a recombination event required evidence from at least two independent methods to be considered.

### 2.6. Data Availability

The sequences derived for this study were deposited into the NCBI database. The accession numbers OK491658-491712, OK539682, and OL856082-92 were assigned.

## 3. Results

### 3.1. Sequencing

The complete coding regions of the 66 SINV isolates derived from the Australasian region were successfully sequenced in the present study. In addition, the complete coding sequence of the Egyptian SINV prototype, AR339, was generated. For all derived sequences, only partial 5′ and 3′ untranslated region (UTR) sequences were resolved, which were trimmed prior to phylogenetic analysis.

### 3.2. Recombination Analysis

Prior to maximum likelihood phylogenetic reconstructions, the complete globally sampled SINV dataset was queried for evidence of recombination. Only isolates H7 and NSV_305 were resolved as recombinants in this analysis, as previously reported, and were excluded from further analysis [19]. There was no evidence of recombination within the newly generated Australasian-derived SINV genome dataset.

### 3.3. Phylogenetic Analysis of Australasian-Derived Sindbis Virus

Two major SINV phylogenetically distinct groups have been described as circulating within Australia, based on previous partial E2 gene sequence analysis. These are the G2 genotype, representing variants in wider circulation throughout Australia, and the G6 genotype, or ‘SW’, which has so far been sampled exclusively from the south-west region of WA. In addition, three Whataroa viruses (WHAV, G5) were detected in eastern Australia between 1989 and 1990, for which only partial sequences are available [22].

**Figure 1:**
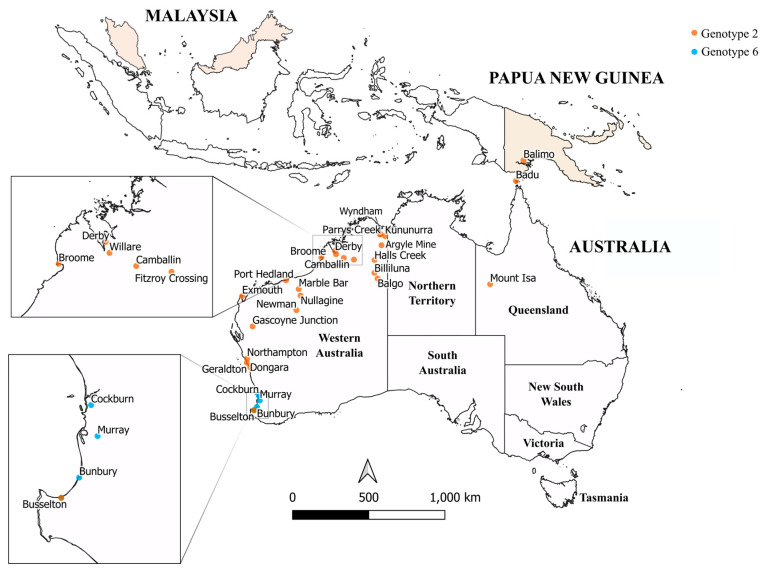
Sampling locations of SINV sequenced for this study, within Papua New Guinea (PNG), Malaysia, and Australia. Genotype 2 and 6 viruses (G2, G6) were both sampled within the region with sampling sites represented by orange and blue markers, respectively. Most Sindbis viruses analyzed in the present study were collected in the Australian state of Western Australia. PNG and Malaysia are shaded in orange to represent sampling of G2 viruses from undefined locations within these countries.

The sampling locations of the viruses sequenced in this study are presented in Figure 1.

The 67 Australasian-derived genome sequences, including the single previously published genome sequence from the region, SW6562, were phylogenetically analyzed using ML phylogenetic reconstruction. SW6562 is the prototype virus of G6 [33].

**Figure 2:**
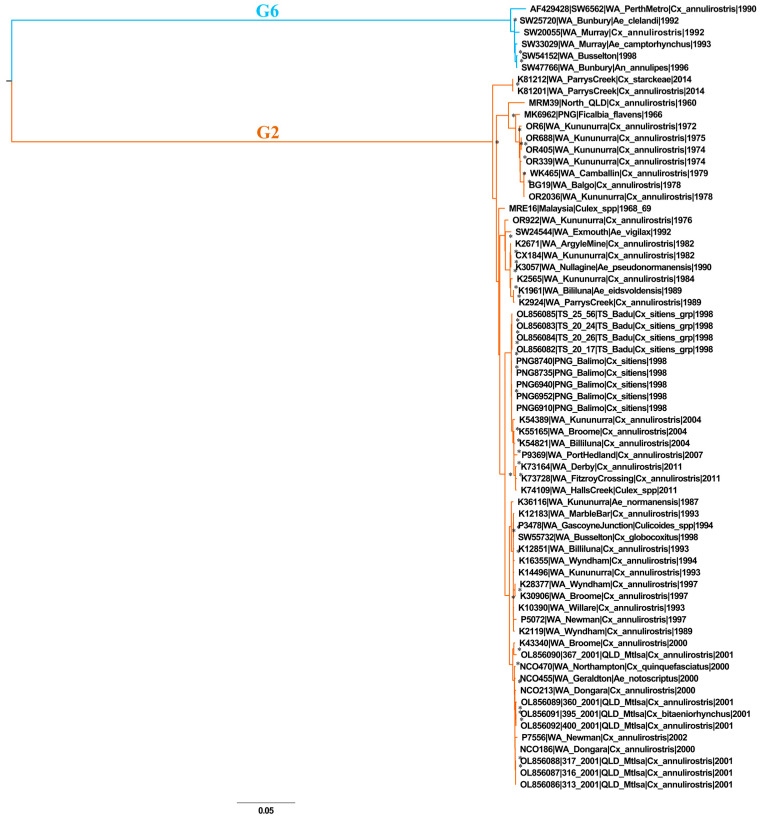
A mid-point rooted maximum likelihood phylogeny of a 67-taxon alignment of Sindbis virus (SINV) sequences sampled from the Australasian region. A single published genome sequence, that of the G6 prototype, SW6562, was included in the analysis. G2 (orange clade) and G6 (blue clade) were both defined in this phylogeny. Asterisks indicate nodes with >80% bootstrap values. Taxon nomenclature details the GenBank accession number, isolate name, sampling location and year of sampling. gp—group, PNG—Papua New Guinea, QLD—Queensland, spp—species, TS—Torres Strait, WA—Western Australia.

Two major genetically divergent groups are defined by the Australasian genome-scale phylogeny (Figure 2). Six of the 67 sequences clustered within SINV G6; all six viruses were sampled exclusively from the south-west region of WA from 1990 to 1998. Despite ample sampling from other locations within WA from 1972 to 2014, G6 viruses were not identified beyond the confined range of the south-west region. The six characterized G6 sequences shared between 97.8 and 99.9%nucleotide sequence identity. No viruses belonging to G5 were sequenced in this collection.

The remaining 61 sequences clustered within G2. Viruses belonging to G2 were sampled from Queensland (QLD) (including the Torres Strait (TS)), Malaysia, Papua New Guinea (PNG), and throughout WA, from 1960 to 2014. Substantial genetic divergence between G2 and G6 was observed, ranging between 25.91 and 26.3% and 14.09 and 14.75% on the nucleotide and amino acid sequence scale, respectively.

**Figure 3:**
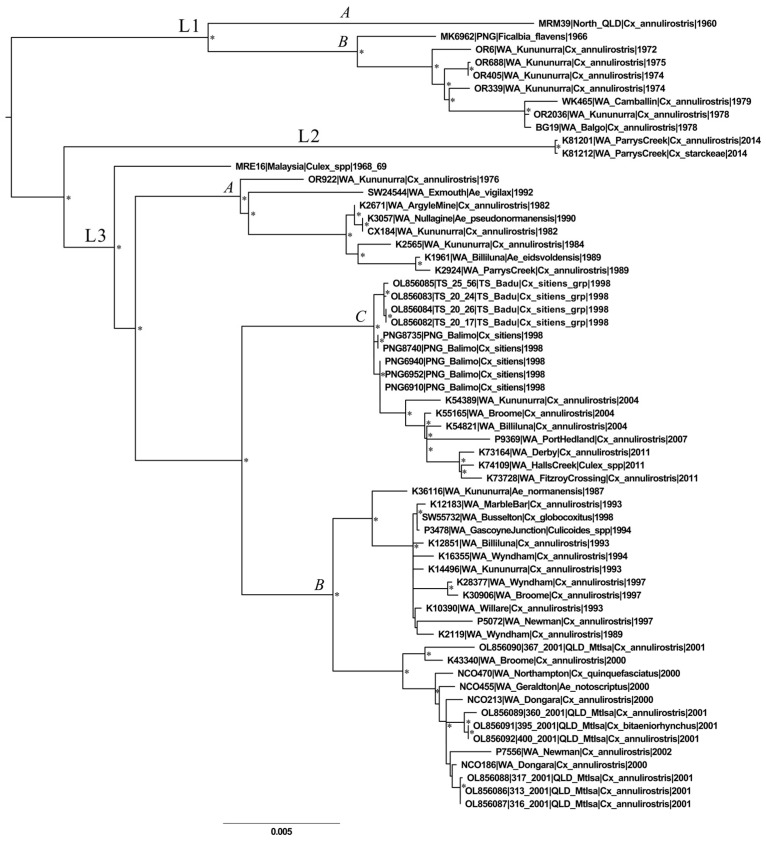
A mid-point rooted maximum likelihood phylogeny of the 61-taxa genotype 2 (G2) Sindbis virus (SINV) dataset. Three distinct lineages (L1–3) were defined in the phylogeny, with minor sub-lineages defined within L1 and L3 (L1A,B, L3A–C). Asterisks indicate nodes with bootstrap values >80%. Taxon nomenclature details the GenBank accession number, isolate name, sampling location and year of sampling. Ae—Aedes, Cx—Culex, grp—group, PNG—Papua New Guinea, QLD—Queensland, spp—species, WA—Western Australia.

To further elucidate genetic variation within G2, an ML reconstruction of the 61-taxa G2 sub-dataset was conducted (Figure 3). Three major groups were defined, lineages 1–3 (L1–3). L1 and L3 each comprised distinct and well-supported sub-lineages (L1A,B, L3A–C). The average pairwise nucleotide sequence divergence (%) between lineages ranged from 2.63 to 3.51% between L2 and L3 and from 3.78 to 4.37% between L1 and L2.

L1 comprised nine isolates sampled from 1960 to 1979 in northern WA, northern QLD, and PNG. The most divergent L1 sequence, MRM39 (sampled in northern QLD, 1960 [55]), demonstrated between 2.4 and 2.9% nucleotide sequence divergence from all the other L1 viruses and comprised its own L1 sub-lineage, L1A. The MRM39 sequence contained a unique 11 amino acid deletion in the nsP3 gene region. The L1B lineage contained isolate MK6962, sampled in the East Sepik region of PNG six years after the sampling of MRM39. MK6962 had a unique single amino acid deletion within nsP3, corresponding to the loss of either a valine or alanine residue. The remaining L1B sub-lineage consisted of isolates sampled in far-north WA from 1972 to 1979.

L2 consisted of just two isolates, both sampled in Parry’s Creek in far north-east WA in 2014. The L2 taxa are terminal to long branches, indicative of the long-term circulation and accumulation of nucleotide changes prior to the sampling of L2. Although it was sampled more contemporaneously, the L2 common node is basal to that of L3. There are two nucleotide differences between the two sampled L2 viruses, which were isolated from two separate mosquito species, *Cx. annulirostris* and *Cx. starckeae*. The L2 viruses were the most contemporary in the dataset. It is not known whether L2 represents what is currently in wider circulation throughout WA, or Australia, or instead represents a low-frequency variant that was sampled serendipitously.

Most of the analyzed sequences clustered within L3, which consisted of viruses sampled from Malaysia, PNG, the TS, QLD, and widely across WA from 1968 to 2011. The lineage demonstrated considerable diversity and comprised three distinct sub-lineages (L3A–C). Malaysian isolate MRE16 had a basal position within L3 and was distinct from the L3A–C viruses. As the MRE16 L3 clade consisted of just a single sequence with no unique features distinguishing it from other L3 sub-lineages, it was not characterized as a distinct L3 sub-lineage. This may change as more archived viruses are sequenced. L3A was diverse, compared with other L3 sub-lineages, and contained isolates sampled over a 12-year period (1976–1990). Interestingly, six viruses sampled in the north of WA were terminal to isolate SW24544, sampled 2–10 years earlier in Exmouth, in the state’s central region of the Gascoyne. SW24544 was up to 1.26% divergent from the six northern isolates at the nucleotide sequence scale, suggesting unsampled diversity in viruses circulating during this period.

L3B consisted of two separate, temporally defined clades. Sampling of the minor L3B sub-clade, from 1987 to 1998, covered a wide geographical span from the south-west north-west and north-east of the state. The sequences of this sub-clade also contained a single aspartic acid deletion, within the nsP3 gene region, not present in any sequence of the other L3B sub-clade. This same amino acid deletion was present in both L2 sequences. Isolate SW55732 is the first characterized G2 SINV virus to be sampled from the south-west of WA—all other SINV sampled from this region have been characterized as G6.

Five PNG-derived sequences, sampled in Balimo from *Cx. sitiens* in 1998, all clustered within L3C and were basal to the WA-derived isolates sampled from 2004 to 2011, sampled from northern and central WA. The TS-derived sequences included in this phylogeny clustered closely with PNG-derived sequences, sampled in the same year, within the L3C sub-lineage. These PNG- and TS-derived sequences are ancestral to the most contemporaneously sampled Australian-derived L3 viruses.

The PNG-derived isolates included in this phylogeny grouped into two separate lineages, L1 and L3, and clustered with Australian-derived sequences collected at similar times. The close spatiotemporal association of PNG- and Australian-derived isolates is indicative of the frequent exchange of virus between the two locations, as opposed to single historic introduction events with subsequent long-term local evolution. This exchange may be facilitated by migratory birds traversing between the two island nations.

Viruses belonging to L1 and L3 appeared to have co-circulated in northern WA during the mid-1970s, as indicated by the sampling of L3 isolate OR922 concurrent with the sampling of L1 viruses from northern WA. Viruses sampled from Mt Isa in north-east QLD in 2001 were diverse, and clustered closely with viruses sampled in northern and central WA in 2000 and 2002.

#### Coalescent Analysis

**Figure 4:**
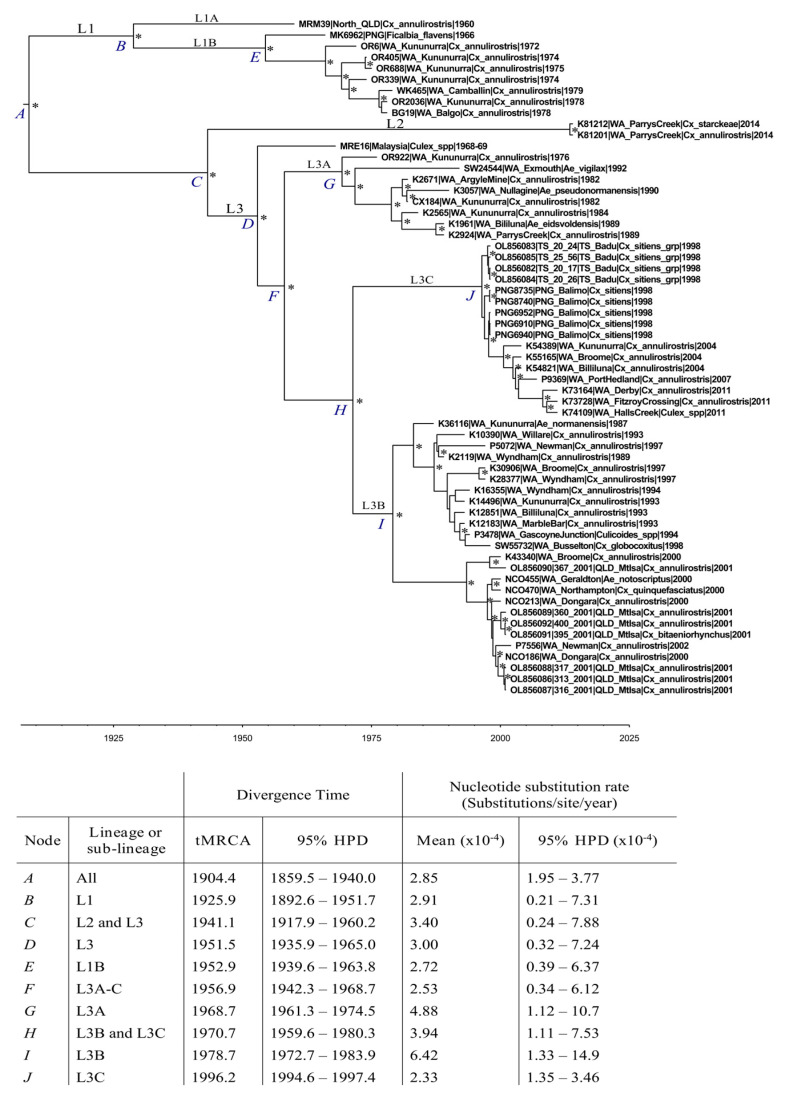
A maximum clade credibility (MCC) reconstruction of the 61-taxa G2 Sindbis virus (SINV) genome sequence dataset, estimated under an uncorrelated log normal (UCLN) molecular clock model, assumed with a GTR + G + I nucleotide substitution model. Posterior probability values of >0.80 are presented above nodes as asterisks. Major nodes are labelled A–I, and are referenced in the lower table, which presents the estimated divergence times (time to most recent common ancestor, tMCRA), and nucleotide substitution rates, with error reported as the 95% highest probability density (95% HPD).

The G2 dataset demonstrated sufficient temporal structure (R^2^: 0.76) for coalescent analyses. A maximum clade credibility (MCC) phylogeny was reconstructed and used to procure temporal estimates, under a UCLN molecular clock assumption (Figure 4).

The time to most recent common ancestor (tMRCA) was estimated as May 1904 (95% highest probability density (HPD): 1859.5–1940.0), or approximately 110 years prior to the most recent G2 sampling in 2014.

The emergence of L1 dates to approximately November 1925 (95% HPD 1892.6–1951.7). Interestingly, the L1A sub-lineage, solely comprising isolate MRM39 (QLD, 1960), was estimated to have emerged approximately 30 years prior to the emergence of L1B, despite just a six-year difference in sampling. This, and the long terminal branch of L1A, suggests the long-term circulation of this lineage prior to its 1960 sampling. Within L1B, the root of the PNG branch (consisting of MK6962) and the Australian-derived branch emerged approximately 13 years apart. L1 has diversified to a limited degree over a prolonged period, based on the available sampling.

The L2 genotype emerged in approximately January 1941 (95% HPD: 1907.2–1960.3), or 73 years prior to the sampling of the two L2 isolates. Both L2 isolates, collected in 2014, sit at the terminus of a long branch, indicating the long-term stability of this lineage over this approximate 70-year period.

The emergence time of L3 was estimated as 1951 (95% HPD: 1935.9–1965.0). There was a 10-year period between the estimated emergence of L2 and L3. The estimated emergence of L3 and L1B overlapped considerably, occurring in approximately 1951 and 1953, respectively (Figure 4). The separate L3 sub-lineages emerged approximately 10–18 years apart. Interestingly, L3A and the common ancestor of L3B and L3C emerged at approximately the same time: in 1969 (mean: 1968.7, 95% HPD: 1961.3–1974.5) and 1971 (mean 1970.7, 95% HPD: 1959.6–1980.3), respectively. L3C is the most contemporaneous L3 sub-lineage, having emerged approximately 26 years ago (mean: 1996.2, 95% HPD: 1994.6–1997.4).

The mean nucleotide substitution rate for G2 was estimated as 2.85 × 10^−4^ (95% HPD: 1.95–3.77 × 10^−4^), equivalent to approximately 3.13 nucleotide substitutions per year. This rate is consistent with, and overlaps with, rate estimates for other alphaviruses, including those circulating within Australia, such as Ross River virus (RRV) (mean rate: 3.21 × 10^−4^, 95% HPD 2.64–3.77 × 10^−4^) and Barmah Forest virus (BFV) (mean rate: 2.56 × 10^−4^, 95% HPD 1.57–4.03 × 10^−4^) [34,56]. These rates were estimated under similar conditions with similarly sized datasets of complete coding sequences. Both RRV and BFV are considered host and vector species generalists.

### 3.4. Phylogenetic Analysis of Australasian Viruses in a Global Context

To understand the genome-scale relatedness of Australasian-derived SINV to SINV sampled on a global scale, a dataset of geographically defined SINV genome sequences was compiled and analyzed. This dataset included AR339, the Egyptian SINV prototype, which was sequenced for this study. Prior phylogenetic analyses of globally sampled SINV have been limited by the prior lack of G2 genotype genome sequences. An ML reconstruction of the globally sampled SINV dataset was conducted, which included 131 complete coding sequences of viruses sampled from 22 separate countries over a 63-year period (1953–2016).

**Figure 5:**
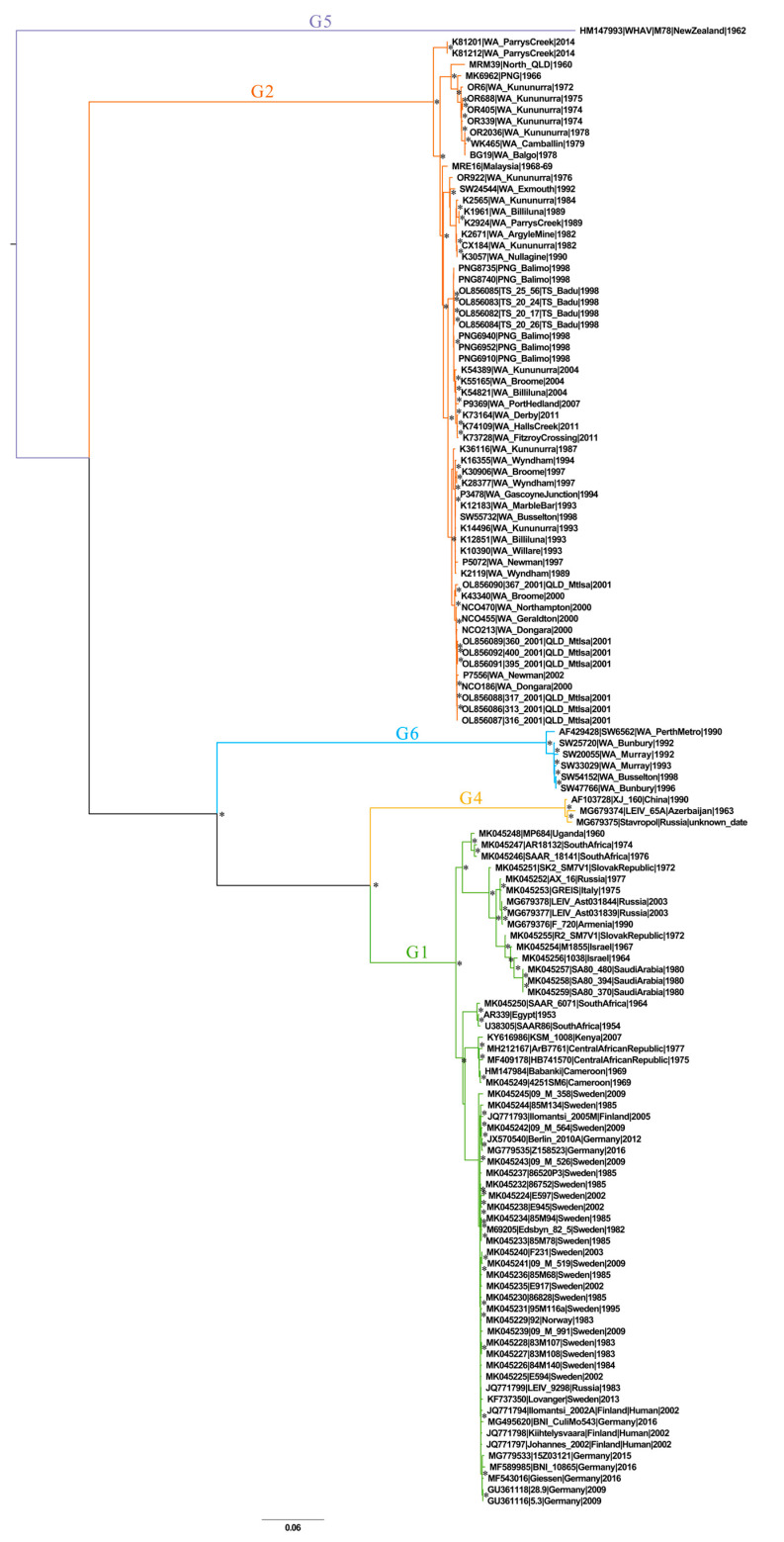
A mid-point rooted maximum likelihood reconstruction of a globally sampled 131-taxa Sindbis virus (SINV) whole genome sequence dataset. Five of the six previously defined SINV genotypes (G1–G6, sans G3) were defined in this genome-scale phylogeny. Asterisks indicate nodes with bootstrap values >80%. WA—Western Australia, QLD—Queensland, PNG—Papua New Guinea, TS—Torres Strait. Taxon nomenclature details the accession number, isolate name, sampling location, and year of sampling.

Five of the six previously defined SINV genotypes (G1-2, 4–6) were resolved in the ML reconstruction (Figure 5). Only partial genome sequences of previously defined G3 viruses were available at the time of this study, and thus were not represented in the genome-scale phylogeny. A supplementary phylogeny was constructed to include the longest G3 sequences (2350 nt), all of which were derived from India (Supplementary Figure S1). All six genotypes (G1–6) were resolved in the supplementary partial genome phylogeny.

#### 3.4.1. Pairwise Nucleotide and Amino Acid Divergence between SINV Genotypes, at the Genome Scale

The average pairwise nucleotide and amino acid sequence distance between each SINV genotype was determined at both the genome scale (Table 1) and partial genome scale of available partial G3 sequences (Supplementary Table S1). The estimated pairwise intergenotype distances presented in both analyses were comparable.

Most genetic groups demonstrated considerable nucleotide and amino acid sequence divergence, ranging from 18.13 to 30.40% and 7.40 to 20.79% divergence, respectively, in pairwise assessment. In contrast, G2 and G3 were separated by at most 9.70% nucleotide and 3.95% amino acid sequence divergence (Supplementary Table S1). Given the low percentages of amino acid and nucleotide sequence divergence existing between G2 and G3, which are comparatively lower than those existing between other genotypes, G2 and G3 will be considered a single diverse genetic group in subsequent analyses. The G2 and G3 group consist of viruses sampled from countries of the wider Australasia region, including India, Australia, Malaysia, and PNG. A single G3 virus has been sampled previously from the Philippines, as defined by a partial genome sequence (<500 nt) that was not included in this analysis. These sampling locations are covered by the East Asia/Australasia migratory flyway zone, with minor flyway routes that indirectly connect between these countries. The combined G2 and G3 dataset, sampled between 1953 and 2014, demonstrated sufficient temporal signal for coalescent analysis. The tMRCA of the dataset was dated to approximately 1764 (95% HPD: 1621.2–1881.7), or approximately 250 years ago. The estimated evolutionary rate of 3.06 × 10^−4^ (95% HPD: 1.92–4.28 × 10^−4^) overlaps with the G2-only rate and corresponds to approximately 3.37 nucleotide substitutions per year.

G5 was the most divergent group of the global dataset, demonstrating between 28.60 and 30.55% nucleotide and 18.98 and 20.79% amino acid sequence divergence from all groups (Table 1). When first isolated, the G5 prototype was referred to as Whataroa virus (WHAV) and noted as having antigenic similarity to SINV, yet sufficient distinction to constitute a unique virus. In the modern literature, WHAV has been referred to as a ‘SIN-like virus’, and in other reports, including the most contemporary SINV phylogeny, as G5 of SINV, despite its large nucleotide and amino acid sequence divergence from other characterizedSINV. WHAV is currently listed as a unique alphavirus species by the ICTV [20].

G4, also previously reported as Kyzylagach virus (KYZV), another ‘SIN-like’ virus, was sampled in central Asian locations including Azerbaijan, western China, and Stavropol in south-west Russia [57,58]. G4 was most closely related to G1 viruses, circulating in nearby western and northern Europe, with a nucleotide and amino acid sequence divergence of 18.13–19.86% and 7.40–8.89%, respectively, at the genome scale. Compared to the other viral groups, G4 demonstrated at least 25.31% and 12.48% nucleotide and amino acid sequence divergence, respectively.

Interestingly, and as previously observed in partial gene sequence analyses, G6 viruses sampled from the south-west of WA were more closely related to G1 viruses derived from Europe and Africa than to G2 viruses sampled proximal to G6 within Australia [16]. G6 viruses have not been detected since 1998, and its origins remain enigmatic.

#### 3.4.2. Comparison of the Largest and Geographically Disparate SINV Genotype Datasets—G1 and G2

G1 and G2 viruses, the most frequently sampled genotypes studied here, demonstrate geographical demarcation as well as large-scale nucleotide and amino acid sequence divergence (Table 1). G1 included viruses sampled exclusively from African and European countries, including locations associated with past SINV disease outbreaks. A supplementary phylogenetic reconstruction was conducted of the 63-taxa G1 dataset, to define the intra-genotype diversity at the genome scale (Supplementary Figure S1), which defined two distinct lineages (L1 and L2). Though sampled over a similar timeframe, and over a similarly large trans-continental geographical span, the intra-genotype diversity of G1 was lower than the diversity observed within the G2/G3 dataset, with maximum nucleotide sequence divergences of 7.35% and 9.7%, respectively.

In contrast to historical views on SINV molecular epidemiology G1 viruses sampled from locations of regular disease outbreaks were not more closely related to each other than to G1 viruses sampled elsewhere (Supplementary Figure S1). On the contrary, these viruses were closely related to viruses sampled in locations not currently associated with outbreaks. The epidemiology of disease, therefore, is likely more complex than previously thought.

Temporal analysis of a G1 sub-dataset, under comparable conditions to the G2 analysis, estimated the evolutionary rate to be 7.36 × 10^−5^ (95% HPD 4.95 × 10^−5^–1.01 × 10^−4^), or approximately 0.81 nucleotide substitutions per year. This rate is approximately 3.87-fold and 4.16-fold lower than the estimated G2 and G2/G3 rates, respectively. The G1 rate estimated here is consistent with the rate reported elsewhere in similar coalescent analyses and is in line with the lower level of diversity accumulated for G1 viruses, compared to G2/G3 viruses in the maximum likelihood analysis [19]. The 95% HPD intervals of this estimate did not overlap with the G2 or the G2/G3 rate estimates, nor those estimated previously for the Australian alphaviruses RRV and BFV [34,56].

## 4. Discussion

Sindbis virus is considered the most widely distributed alphavirus species, with documented sampling from multiple countries in Europe, Africa, and Australasia. The definition and nomenclature for SINV genetic groups are inconsistent between studies and have been limited by partial gene sequence analyses. Available SINV sequences and datasets mostly comprise viruses derived from Europe and Africa, where disease outbreaks have been reported [4,7]. The most recent SINV phylogenetic review [7] defined six unique SINV genotypes, G1–G6, sampled globally. Prior to our investigation, only a single SINV full genome sequence from the Australasian region was available, which belonged to the G6 prototype. In the present study, genome sequence representation of the Australasian region was greatly expanded, with the assembly of 66 new whole genome sequences that were sampled from three countries over a 54-year time span. This allowed for a more robust, genome-scale phylogenetic analysis of globally sampled SINV, and characterized the extent of divergence between the G1–G6 groups for the first time. Here, these six groups were found to be highly disparate at the nucleotide and amino acid scales.

Analysis of the diversity of circulating SINV in Australia has been restricted to a single, partial gene sequence analysis of historical isolates that defined two broad groups in circulation, currently referred to as G2 and G6. Our analysis confirmed G2 to be the dominant SINV group that continues to circulate within Australia. We defined three distinct lineages of G2, lineages 1–3, sampled between 1960 and 2014 from PNG, Malaysia, and Australia. Temporal and genetic analysis of PNG- and Australian-derived SINV indicated potential intermittent exchange of viruses between the two regions, likely facilitated by migratory birds. This contrasts with the previously observed patterns of long-term local evolution following historical exchange that have been documented for other Australian alphaviruses, Ross River virus (RRV) and Barmah Forest virus (BFV) [56,59,60]. Genetic homogeneity of G2 viruses sampled over a wide geographical range in a narrow period was also observed. In agreement with previous analyses, the sampling of G6 was restricted to the south-west region of WA, despite extensive sampling from other regions over time [16].

Genome-scale analysis of globally sampled SINV defined large-scale sequence divergence between most of the previously described SINV genotypes (G1–G6, sans G3). All groups, except for G1 and G4, demonstrated at least 23% and 10% nucleotide and amino acid sequence divergence, respectively, at the genome scale. Full-length G3 genome sequences were not available for analysis in this study; however, demarcation of a similar scale was observed between the SINV groups when analyzed on the partial genome, 2000 nt scale, of the available G3 sequences, except between the G3 and G2 viruses, which were both sampled within the wider Australasian region.

The 9th International Committee on Taxonomy of Viruses (ICTV) *Togaviridae* report defines alphavirus species demarcation as demonstrating at least 23% and 8% nucleotide and amino acid sequence divergence, respectively [61]. These criteria are satisfied by most of the SINV genetic groups analyzed in this study. This degree of divergence is observed between the G1 SINV groups (containing the AR399 SINV prototype) and the G2, G5, and G6 genetic groups, and thus each group could be considered unique viral species based on these criteria alone. The genetic divergence observed between G2 and G3, at the partial genome scale, does not satisfy these criteria, and thus these viruses may be considered a single, highly diverse genetic group.

The more recent 10th ICTV report expands these criteria, requiring additional evidence of human pathogenic, antigenic, and ecological differences to define unique species [20]. The phenotypic properties of SINV circulating beyond Europe and Africa are largely uncharacterized. However, G2 and G6 are recognized to cause largely inapparent infection with only two SINV cases ever described in Australia, which were diagnosed serologically in areas of high endemic alphavirus prevalence [9,14]. Further studies will be undertaken to define the antigenic features of G2 and G6 viruses.

The G5 viruses were the most disparate genetic group characterized in this study, with the nucleotide and amino acid sequence divergence ranging from 28.6 to 30.55% and 18.98 to 20.79% between all groups, respectively. When the G5 prototype M78 was first isolated from *Cx. pervigilans* mosquitoes in 1962, the isolate was noted to have antigenic similarity to the SINV prototype, yet was sufficiently antigenically and genetically distinct to be characterized as a unique viral species, Whataroa virus (WHAV) [21,62,63]. SINV studies in subsequent years referred to WHAV as a ‘SIN-like’ virus, and then as the fifth genotype of SINV, including in the most recently published phylogenetic review [7]. WHAV is defined in the 10th ICTV *Togaviridae* report as a unique alphavirus species, a distinction that is supported by the large-scale sequence divergence observed in the present study and by historically documented antigenic variation [21,22]. WHAV should no longer be referred to as a SIN-like virus, nor as a unique SINV genotype.

The six G6 genome sequences analyzed in this study were derived from mosquitoes trapped in the south-west region of WA over a narrow eight-year sampling period. Despite extensive sampling from throughout WA over time, no other G6 viruses have been identified beyond this area. This geographical restriction may indicate that these viruses are maintained in a transmission cycle involving a more sedentary host than the postulated avian hosts of SINV [16]. Interestingly, despite the geographical proximity of G2 and G6 sampling, these viruses are highly genetically disparate. G6 viruses were more closely related to G1 viruses sampled in Africa and Europe, a zoogeographical region that shares no overlapping major avian flyway coverage with Australia [17,64]. The origins of G6 remain ambiguous.

In comparing G1 and G2, which are now the best represented SINV genotypes in terms of genome sequence availability, several clear differences were noted. G1 and G2, as well as other SINV groups, demonstrate clear geographical and genetic demarcation. No Sindbis disease outbreaks have been reported in the Australasian region where G2 is dominant, in contrast to the African–European region where G1 viruses circulate. The temporal analysis presented here indicates that G1 viruses have evolved at a slower rate compared to the sampled G2/G3 population, with up to 4.1-fold fewer nucleotide substitutions per year. These rate differences may reflect differential ecological pressures and niches occupied by these two distinct virus groups. Nominal evidence of antigenic demarcation between Australian- and European/African- derived SINV was described in a 1985 study by Olson and Trent where viruses from Australia and India (G2 and G3 viruses) did not react with an anti-AR339 (G1) E2 monoclonal antibody to significant titers [65]. We propose that G2 and G3 viruses should be collectively considered a unique virus species, named Argyle virus (ARGV), based on the observed genetic divergence and nominal evidence of antigenic, pathogenic, and ecological differences from SINV (G1) [65]. Similarly, we propose that G6 viruses be considered a unique viral species named Thomson’s Lake virus (THLV) as supported by large-scale genetic divergence and divergent ecology from SINV and ARGV, based on the clear geographical restriction of THLV.

The G4 viruses included in this analysis were sampled in central Asia and eastern Europe. These viruses demonstrated between 18.13 and 19.86% and 7.4 and 8.89% divergence at the nucleotide and amino acid sequence scales, respectively—insufficient, alone, to demarcate a distinct species from SINV using the ICTV genetic criteria [61]. G4 could be considered a highly disparate distant genetic group of SINV.

Further studies should be undertaken to define the pathogenic and antigenic properties of ARGV (G2 and G3) and THLV (G6) compared to SINV (G1 and G4) and WHAV (G5). The antigenic relationships should be assessed using monoclonal antibodies specific to each virus group and human antisera from natural infections in South Africa or northern Europe, the only regions where human disease is well characterized, to inform pathogenicity studies in animal models.

## 5. Conclusions

We propose the designation of genotype 6 (G6) and genotypes 2 and 3 (G2 and G3) as Thomson’s Lake virus (THLV) and Argyle virus (ARGV), respectively.

This is based on the large-scale nucleotide (>23.2%) and amino acid (>10.3%) sequence divergence identified in our analysis of Australasian ‘Sindbis’ viruses, compared to SINV G1 and G4 viruses. In addition to this genetic divergence, ARGV and THLV are geographically, ecologically, and evolutionarily distinct and are not associated with human disease.

## Figures and Tables

**Table 1:** The percentage pairwise distance ranges (%) between the defined genotypes (G1,2, G4–6) of SINV, at the nucleotide (upper quadrant) and amino acid (lower quadrant) sequence scales, across the entire coding region. The genotype names in the table headers are coloured to correspond with the colours employed to label these genotypes in Figure 5.

	**G1**	**G2**	**G4**	**G5**	**G6**
**G1**		25.26–26.83	18.13–19.86	28.60–29.85	23.15–24.70
**G2**	13.03–14.55		26.60–27.06	29.97–30.55	26.03–26.65
**G4**	7.40–8.89	14.45–15.24		30.37–30.40	25.31–25.68
**G5**	18.98–20.00	20.01–20.41	20.21–20.46		29.61–30.05
**G6**	10.28–12.61	14.16–15.75	12.48–13.89	19.73–20.79	

## Data Availability

Data presented in this article are available in the article.

## Supplementary Materials

The following supporting information can be downloaded at https://www.mdpi.com/article/10.3390/v16010007/s1, Figure S1: Maximum likelihood reconstruction of a globally sampled Sindbis virus partial genome sequence dataset, including G3 sequences; Table S1: Pairwise nucleotide and amino acid distances between SINV genotypes, G1–G6, at the partial genome scale.
