# Supplementary material for: Genomic Analysis of Sindbis Virus Reveals Uncharacterized Diversity within the Australasian Region, and Support for Revised SINV Taxonomy
Source: Viruses. 2023 Dec 20;16(1):7. doi: 10.3390/v16010007 (PMC10820390; doi:10.3390/v16010007)
Supplement: Supplementary file 1 [file viruses-16-00007-s001.zip › Supplementary/Supp_Tab1_mk2_newblue.pdf]

	G1	G2	G3	G4	G5	G6
G1		24.50– 26.84	24.52 – 26.24	17.82 – 19.91	28.37 – 29.39	22.50 – 24.25
G2	13.67 – 16.60		8.55 – 9.70	26.93 – 27.95	30.20 – 31.26	25.90 – 27.52
G3	14.18 – 16.09	1.53 – 3.95		26.58 – 27.52	30.20 – 30.31	26.24 – 27.39
G4	9.45 – 11.49	16.22 – 18.14	16.47 – 17.11		30.54 – 30.63	26.54 – 26.93
G5	21.07 – 21.97	22.61 – 23.63	23.12 – 23.24	22.35 – 22.73		29.09 – 29.48
G6	12.01 – 14.05	15.33 – 17.63	16.22 – 17.62	15.07 – 16.60	21.84 – 22.73	
