# Supplementary figures and images for: Genomic Analysis of Sindbis Virus Reveals Uncharacterized Diversity within the Australasian Region, and Support for Revised SINV Taxonomy
Source: Viruses. 2023 Dec 20;16(1):7. doi: 10.3390/v16010007 (PMC10820390; doi:10.3390/v16010007)

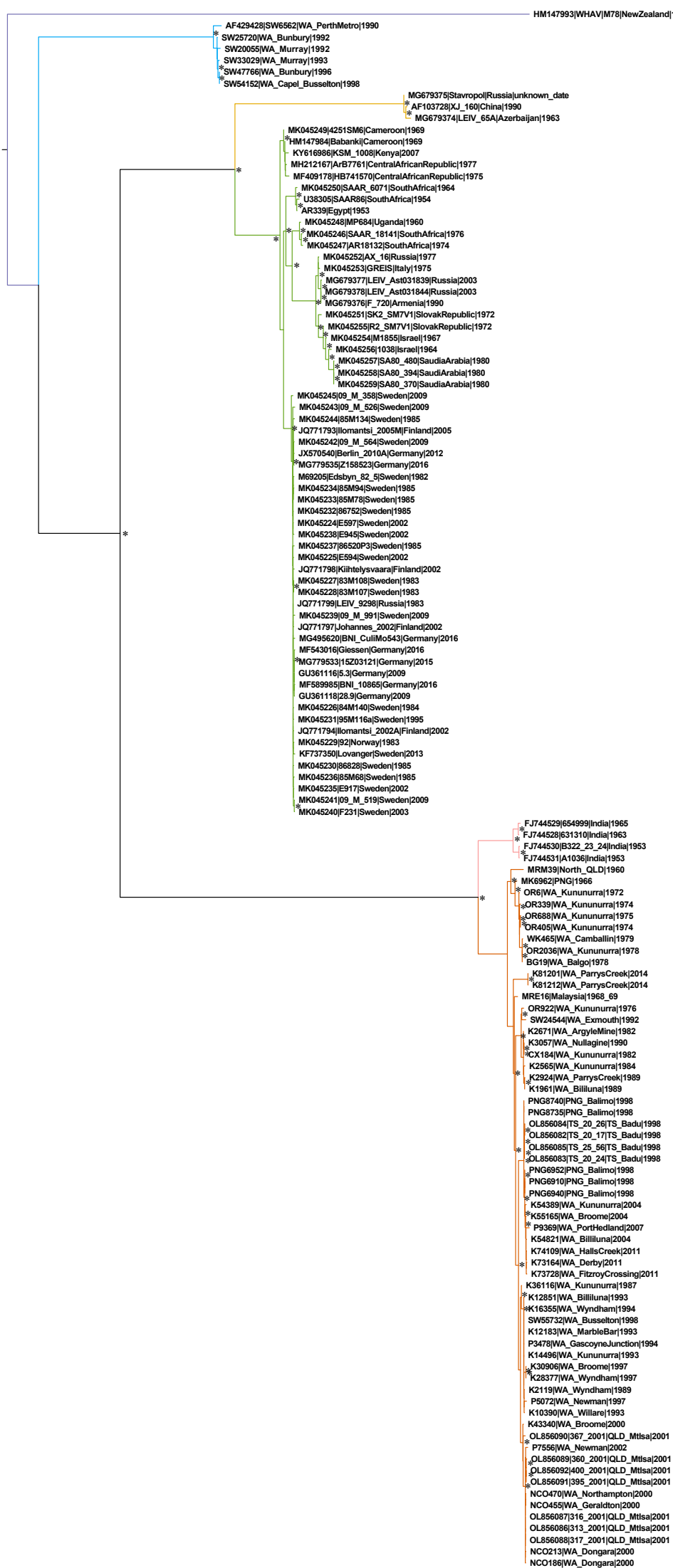

Supplement: Supplementary file 1 [file viruses-16-00007-s001.zip › Supplementary/Supp_Fig1_135tML_mk3_newblue.pdf]

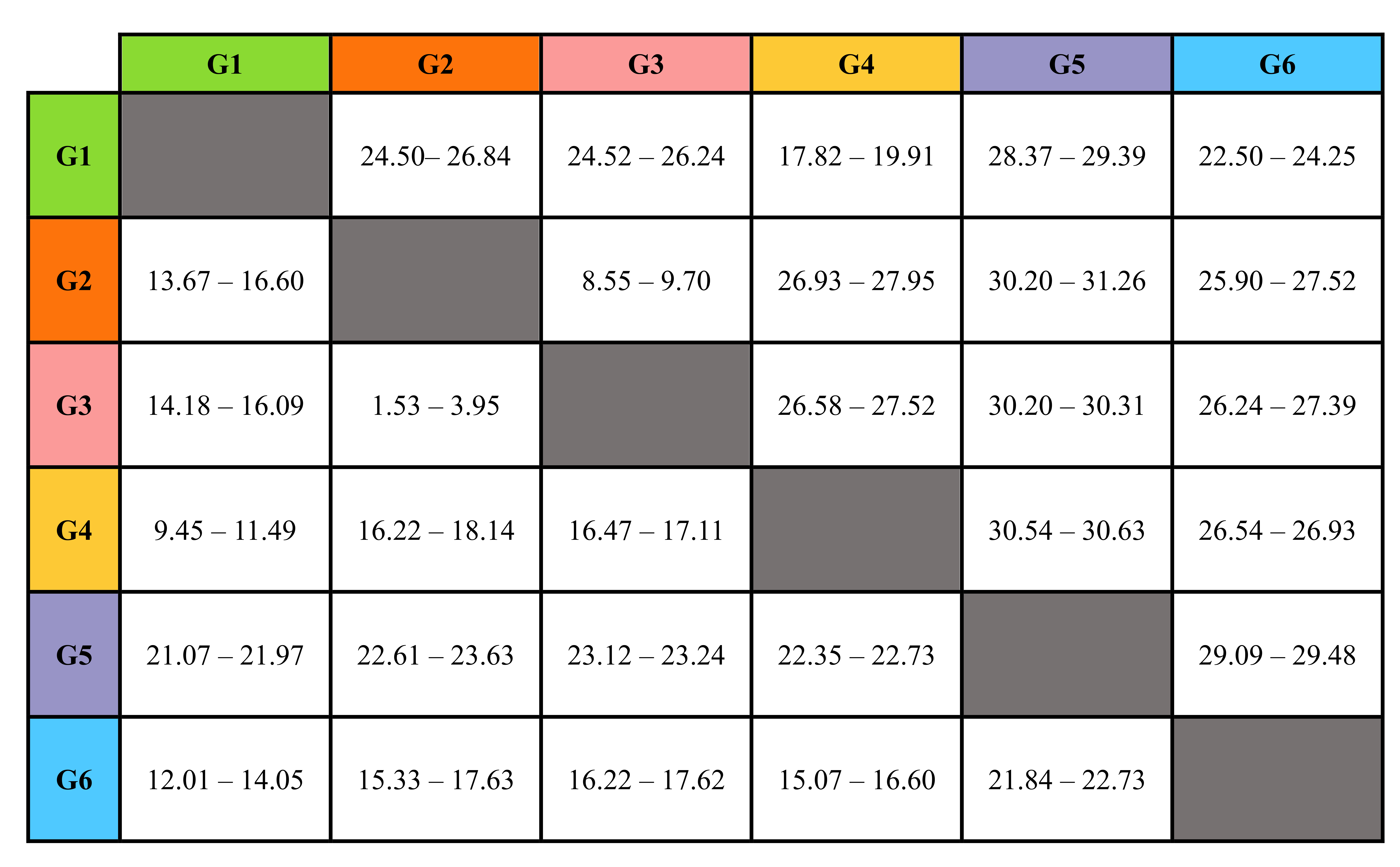

Supplement: Supplementary file 1 [file viruses-16-00007-s001.zip › Supplementary/Supp_Tab1_mk2_newblue.png]
